# Cardiac sympathetic denervation for catecholaminergic polymorphic ventricular tachycardia in the light of the medical situation in Japan

**DOI:** 10.1002/joa3.13069

**Published:** 2024-05-20

**Authors:** Masayoshi Mori, Hisaaki Aoki, Kumiyo Matsuo, Dai Asada, Yoichiro Ishii

**Affiliations:** ^1^ Department of Pediatric Cardiology Osaka Women's and Children's Hospital Osaka Japan

**Keywords:** aborted cardiac arrest, cardiac sympathetic denervation, catecholaminergic polymorphic ventricular tachycardia, neuropathic pain, ptosis

## Abstract

Progress of treadmill exercise testing in Case 1 Each electrocardiogram shows the maximum load. Before left cardiac sympathetic denervation, polymorphic ventricular tachycardias were observed. After left cardiac sympathetic denervation, no ventricular arrhythmias were induced during exercise.

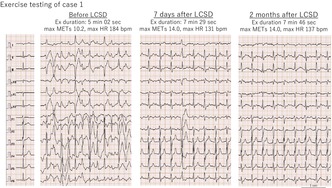

Catecholaminergic polymorphic ventricular tachycardia (CPVT) is a severe arrhythmia that can induce ventricular tachycardia (VT) and ventricular fibrillation (VF), leading to fatal outcomes during physical activity or emotional stress. In cases where pharmacological treatment (e.g., beta‐blockers or flecainide) is ineffective, other treatment options include left cardiac sympathetic denervation (LCSD) and an implantable cardioverter‐defibrillator (ICD). Although ICD has been performed during resuscitation, there have been reports of worsening arrhythmia and mortality following ICD defibrillation.^1^ Thus, the indications for ICD should be approached with greater caution. LCSD for CPVT reportedly reduces cardiac events from 100% to 32% and decreases the shock activation rate in patients with ICDs by 93%.^2^ The effects of LCSD include prolonging ventricular refractoriness, thereby reducing the likelihood of reentrant arrhythmia, and the reflexive increase in cardiac vagal efferent activity. However, LCSD is not frequently performed in Japanese individuals with CPVT, and a possible contributory factor is the lack of health insurance coverage in Japan. Two cases of cardiac sympathetic denervation for CPVT were reported, wherein the procedure was found to be effective in managing ventricular arrhythmias. Case 1: A 14‐year‐old boy has experienced syncopal episodes during exercise since he was 7 years old. He was diagnosed with CPVT at 9 years of age because a treadmill exercise test revealed bidirectional VT. This was managed with exercise restriction and nadolol (1 mg/kg/day), and he has not experienced any syncopal episodes since then. The family history was negative for syncope, sudden cardiac death, and ryanodine receptor (RyR2) gene mutations. In our case, only the RYR2 gene has been examined, and consent for genetic testing of both parents and siblings could not be obtained. There is no family history of consanguinity. The patient experienced aborted cardiac arrest with VF during jogging, with no neurological sequelae. After obtaining ethical approval, the patient was managed with thoracoscopic LCSD (Figure 1) and flecainide. Exercise testing revealed that premature ventricular contraction (PVC) and VT were not induced. The Holter electrocardiogram exhibited no PVCs, confirming the effectiveness of LCSD (Figure 2, Table 1). He presented with transient sweating pattern changes, neuropathic pain, and mild left upper ptosis (Figure 3A); however, facial flushing was not observed. Case 2: A 14‐year‐old boy who had undergone percutaneous transluminal pulmonary valvuloplasty for pulmonary valve stenosis developed bradycardia, left bundle branch block, exercise‐induced atrial tachycardia, and PVC during the follow‐up period. He had several febrile seizures, but no syncopal episodes. He was diagnosed with CPVT at 8 years of age, because exercise testing revealed bidirectional VT. Holter ECG showed total heartbeats of 100,965 beats, and a heart rate (HR) (min/ave/max) of 47/73/175 bpm. The patient was managed with exercise restriction and nadolol (0.5 mg/kg/day) and flecainide (2.5 mg/kg/day). RyR2 gene mutations (c.5128C>A, p. H1710N) were present in the patient and his mother. At the age of 14 years, he experienced cardiac arrest while watching a baseball game, and five defibrillations were performed because of VF. There were no neurological sequelae. Our case has monomorphic PVCs and PACs. When implanting the ICD, ablation was performed to reduce the risk of VF storm and inappropriate ICD functioning because of atrial tachycardia. However, during the attempt to induce PVC, a small amount of isoprenaline was administered. The PVC quickly became polymorphic, resulting in the induction of VF and the subsequent termination of the procedure. He underwent thoracoscopical LCSD because catheter ablation was ineffective for AT and VT. One week after LCSD, there was a slight decrease in the inducibility of arrhythmias, such as polymorphic VT and AT. Six months after LCSD, right cardiac sympathetic denervation (RCSD) was performed because the inducibility of PVCs and AT worsened, although the patient did not experience any syncopal episodes. One month after RCSD, exercise testing revealed improved inducibility of arrhythmia, while Holter ECG revealed the following: Total heart beats of 62,715 heart rate (minimum/average/maximum) of 30/44/98 bpm, PVC 667 beats (1%), and nonsustained AT (26 beats) and VT (7 beats). ICD implantation was deferred because he had general fatigue because of bradycardia and a history of aborted cardiac death despite bilateral cardiac sympathetic denervation (CSD). Four months after RCSD, exercise testing revealed more improved inducibility of arrhythmia (Figure 4, Table 1). Following cardiac sympathetic denervation, there was a decrease in chronotropic action during exercise, resulting in a decreased rate response during exercise. He had dry skin in both upper extremities and transient neuropathic pain, but no facial flushing or hand numbness. Ptosis was prominent after RCSD, but not after LCSD; this improved 2 months after RCSD (Figure 3B). LCSD was effective in Case 1, whereas RCSD followed by LCSD was effective in Case 2. Although LCSD is recommended in long QT syndrome and CPVT according to the ESC and HRS guidelines, reports on this are rare in Japan.^3^ Whenever syncope occurs despite optimal medical therapy, LCSD could be considered the next step rather than an ICD and could complement ICDs in patients with recurrent shocks. Bilateral CSD should be performed for intractable VT in patients with hereditary arrhythmia as well as structural heart disease (e.g., dilated cardiomyopathies and ischemic heart disease). Okajima et al. reported that RCSD followed by LCSD was effective in CPVT patients who experienced frequent ICD shocks related to VF or AT with rapid ventricular response.^4^ Meanwhile, Miki et al. reported that bilateral CSD suppressed VTs in the acute phase in patients with heart failure with reduced ejection fraction.^5^ The complications of LCSD include ptosis, neuralgia, and palmar anhidrosis. In our study, patients had only transient or mild symptoms that were well tolerated. Nevertheless, the efficacy of CSD in hereditary arrhythmias requires further validation in more cases. In the future, CSD for severe arrhythmias will hopefully be covered by Japanese health insurance.

**FIGURE 1:**
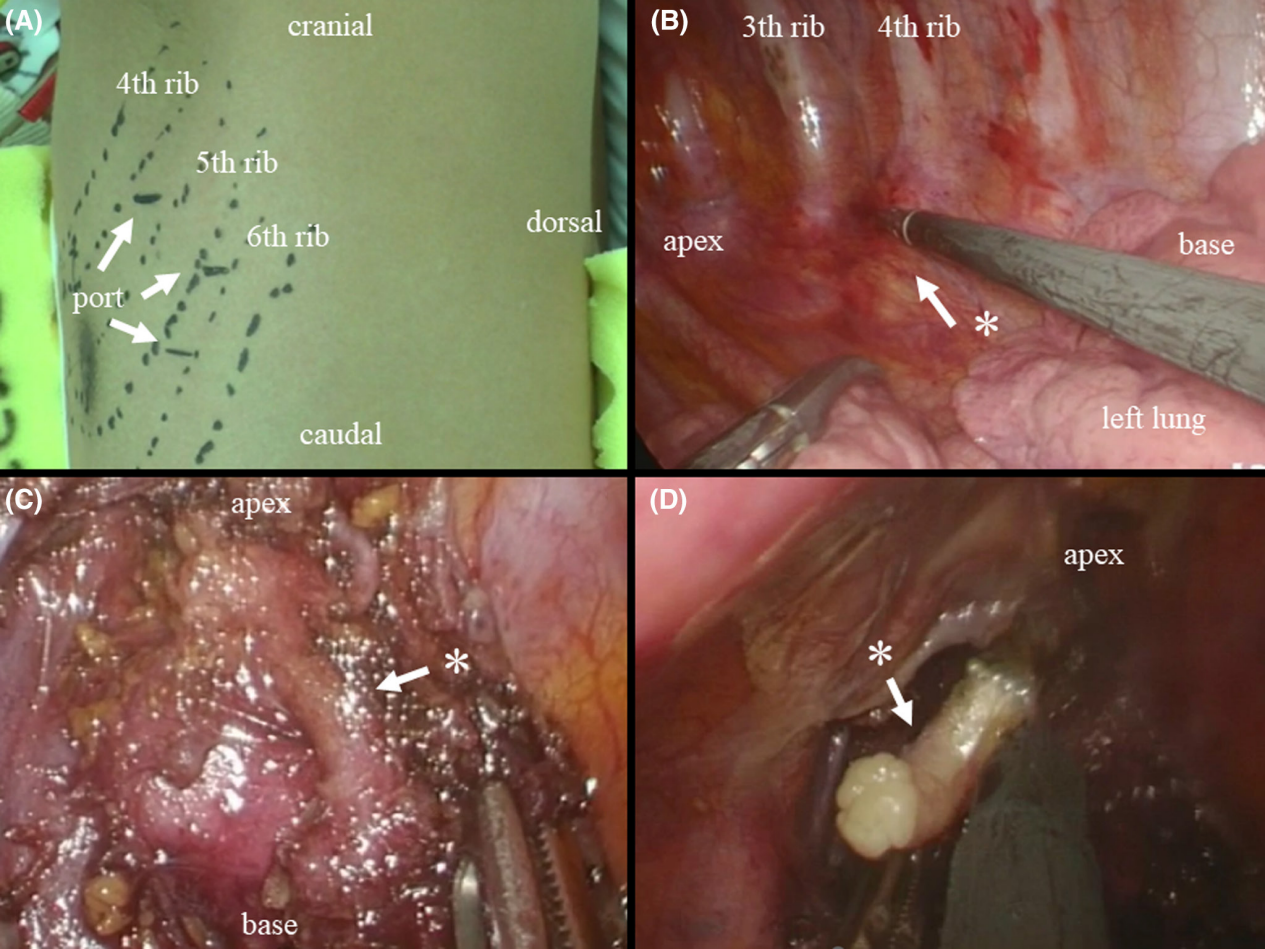
(A) Surgical procedure for left cardiac sympathetic denervation: Three ports were inserted along the fourth intercostal space anterior axillary line, the fifth intercostal space midaxillary line and seventh intercostal space posterior axillary line. (B–D) The left sympathetic nerve (*) from T2 to T4 and the stellate ganglion (T1) were identified, and the stellate ganglion was dissected at the lower half.

**FIGURE 2:**
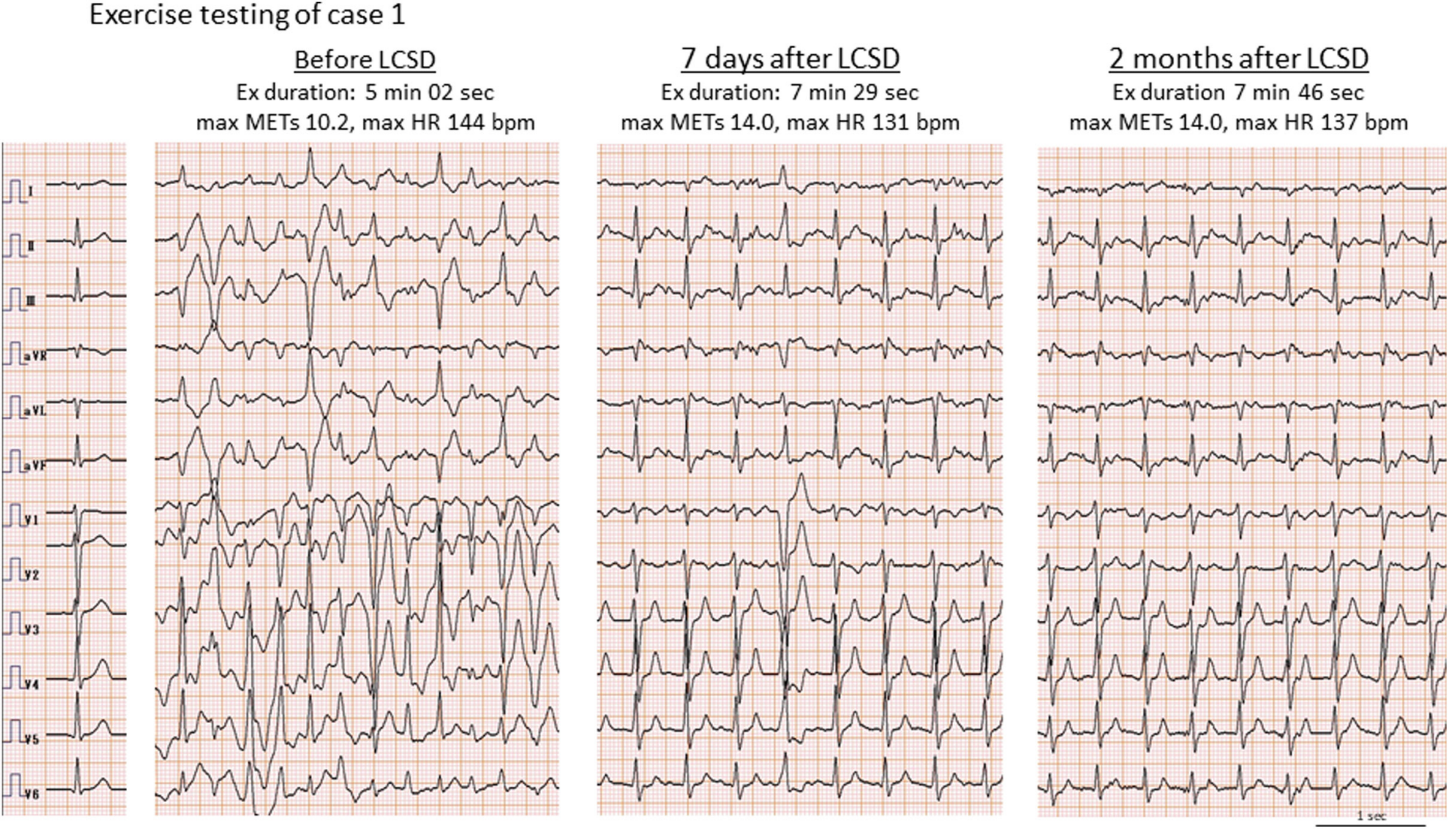
The treadmill exercise testing in Case 1 Each electrocardiogram shows the maximum load. The treadmill stress tests were conducted until leg fatigue or dyspnea reached maximal levels, or when the frequency of PVCs increased. Before LCSD, polymorphic VTs were observed. After LCSD, no ventricular arrhythmias were induced during exercise. After LCSD, there was a reduction in the rise in heart rate during exercise. Ex, exercise; HR, heart rate; LCSD, left cardiac sympathetic denervation; METs, metabolic equivalents.

**TABLE 1:** Summary of Exercise testing and Holter ECG.

		Case 1			Case 2		
		Pre 13 years old	7 days after LCSD	2 months after LCSD	Pre 8 years old	5 months after LCSD	4 months after RCSD
<Tredmill>	Max HR	184	131	137	144	126	127
	Ex duration	5 min 02	7 min 29 s	7 min 46 s	8 min 16 s	4 min 20 s	6 min 26 s
	Max METs	10.2	14	14	14	10	14
	Total PVC	263	1	0	534	72	0
	VT	Induced	None	None	Induced	None	None
<Holter>	THB	91,624	110,366	108,449	100,965	69,586	A pacing 98,766
	Min‐max(ave)	44–128 (65)	61–109 (77)	51–111 (76)	47–175 (73)	34–93 (49)	58–104 (70)
	PAC/day	0	0	0	936	65	6
	PVC/day	2	0	0	6659	517	5
	VT	None	None	None	Induced	None	None

Abbreviations: A pacing, atrial pacing; Ex, exercise; HR, heart rate; LCSD, left cardiac sympathetic denervation; METs, metabolic equivalents; PAC, premature atrial contraction; PVC, premature ventricular contraction; RCSD, right cardiac sympathetic denervation; THB, total heart beats; VT, ventricular tachycardia.

**FIGURE 3:**
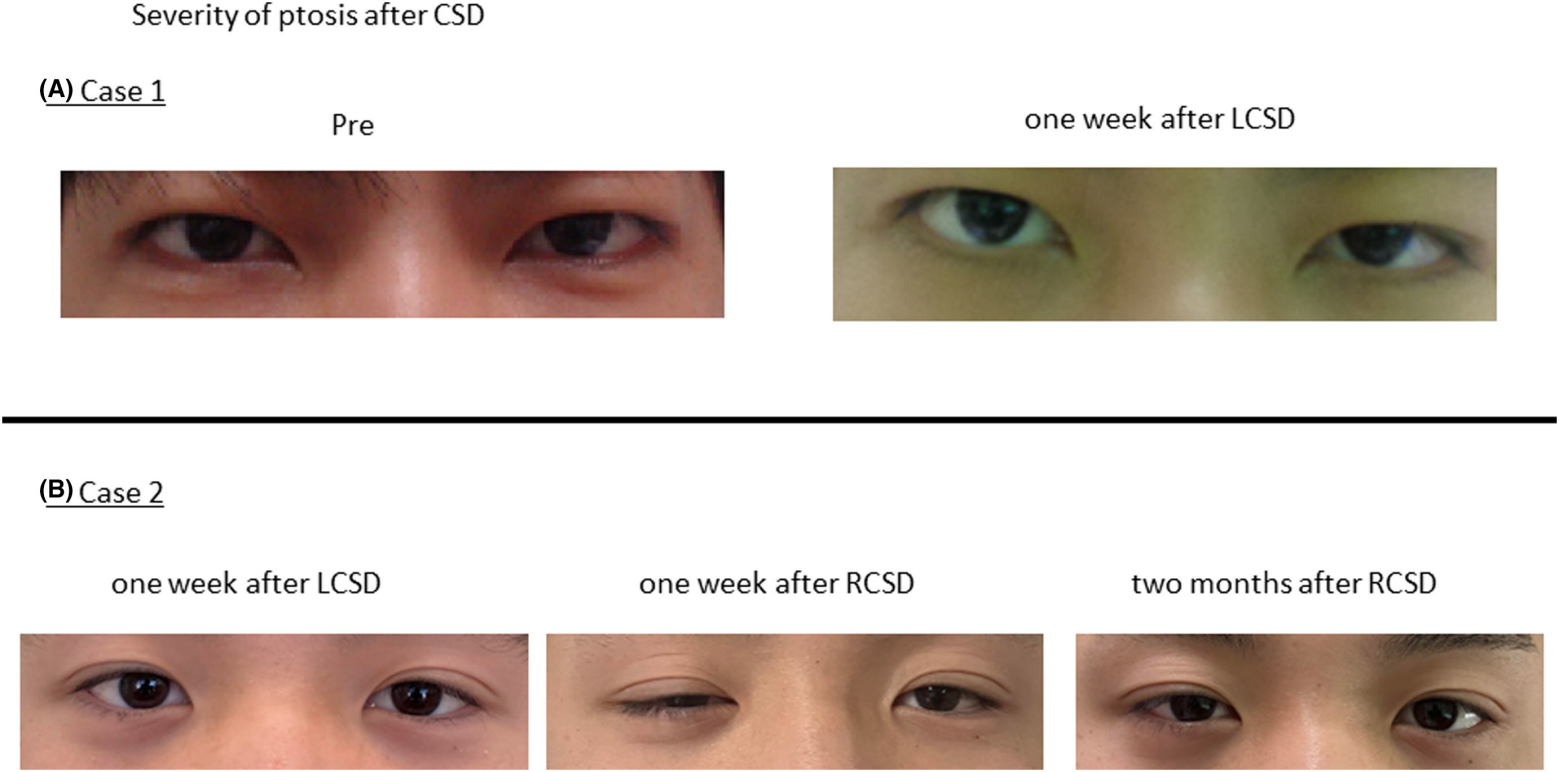
Degree of ptosis before and after sympathetic denervation. CSD, cardiac sympathetic denervation; LCSD, left cardiac sympathetic denervation; RCSD, right cardiac sympathetic denervation.

**FIGURE 4:**
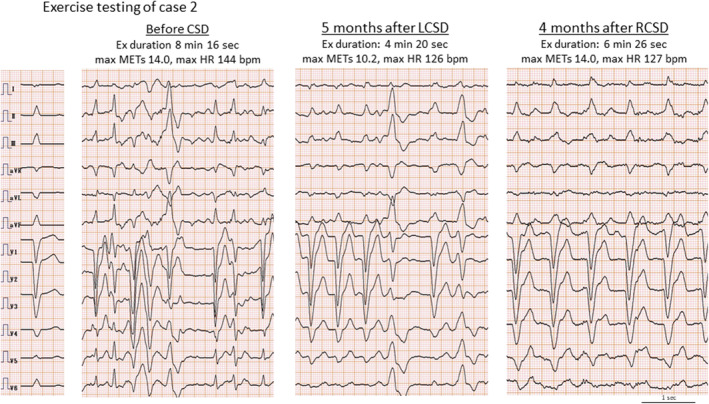
The treadmill exercise testing in Case 2 Each electrocardiogram shows the maximum load. The treadmill stress tests were conducted until leg fatigue or dyspnea reached maximal levels, or when the frequency of PVCs increased. Before LCSD, polymorphic VTs were observed. After LCSD, the inducibility of the arrhythmias decreased, but AT and polymorphic PVCs were still observed. After RCSD, the inducibility of AT and PVCs decreased. After cardiac sympathetic denervation, there was a reduction in the rise in heart rate during exercise. Ex, exercise; HR, heart rate; LCSD, left cardiac sympathetic denervation; METs, metabolic equivalents; RCSD, right cardiac sympathetic denervation.

## FUNDING INFORMATION

This research did not receive any specific grant from funding agencies in the public, commercial, or not‐for‐profit sectors.

## CONFLICT OF INTEREST STATEMENT

Authors declare no conflict of interests for this article.

## ETHICS STATEMENT

This study has received approval from the ethics committee of Osaka Women's and Children's Hospital (number: 1633,1676) and is considered exempt from requiring written consent.

## PATIENT CONSENT STATEMENT

The patients have provided consent for publication.

## Supporting information


**Figure S1:** Family history.
